# Estimating Concentration Response Function and Change-Point using Time-Course and Calibration Data

**Published:** 2019-03-18

**Authors:** B Qiang, A Abdalla, S Morgan, P Hashemi, E Peña

**Affiliations:** 1 Department of Math and Stats, Southern Illinois University Edwardsville, USA; 2 Department of Chemistry and Biochemistry, USC, Columbia, SC 29208; 3 Department of Statistics, USC, USA

**Keywords:** Calibration study, Change-point, General linear model, Chemical concentration, Time-course experiment

## Abstract

In this paper the problem of determining the functional relationship between time and the concentration of a chemical substance is studied. An intervention drug is administered on the experimental unit from which the chemical substance (specimen) is measured. This drug is hypothesized to cause a change in the concentration level of the chemical substance a certain lag-time after the intervention. However, the concentration value could not be directly measured, but rather a surrogate response can be measured. In the time-course study, this surrogate response is measured using different electrodes which possess varied behaviors. To utilize these surrogate measurements arising from the different electrodes (sensors), a calibration study is undertaken which measures the surrogate response for the different electrodes at known concentration levels. Based on the time-course and calibration data sets, a statistical procedure to estimate the signal function and the lag-time is proposed. Simulation studies indicate that the proposed procedure is able to reasonably recover the signal function and the lag-time. The procedure is then applied to the real data sets obtained during an analytical chemistry experiment.

## Introduction

The general problem tackled in this paper is determining the time-behavior of the concentration of a chemical obtained from an experimental unit (e.u.) subjected to an intervention. Specifically, of interest is to study if a specific drug intervention causes a change in concentration. Concentration levels, however, are difficult to measure directly, especially when the e.u. could not be sacrificed, such as during a time-course study. Thus, the concentration levels are to be inferred indirectly through electric charge measurements which are stochastically related to the concentration levels. The measurement of the charge is reliant on the use of an electrode. But different electrodes possess different behaviors when measuring the charge. Thus, to be able to use different electrodes to infer the concentration levels, a calibration study using these electrodes is performed to determine the relationship between concentration and charge for each of the electrodes.

**Figure 1:**
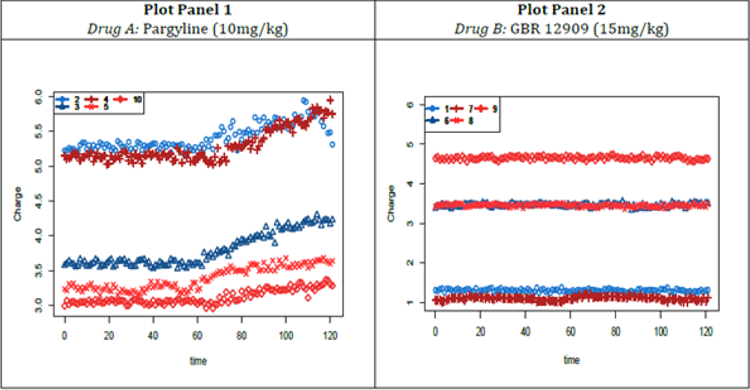
Charge measurements (in pC) on the specimens obtained in the time-course study associated with 5 electrodes over time (in minute) for each of the two drug interventions. Plot panel 1 is for the drug pargyline, while plot panel 2 is for the drug GBR 12909. Different symbols/numbers and colors correspond to different measuring electrodes. Adapted with permission from [1]. Copyright 2017 American Chemical Society.

**Figure 2:**
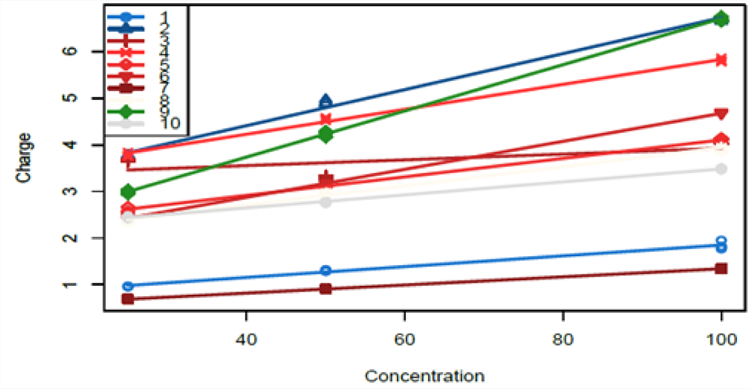
Concentration (in *nM* ) and charge (in *p^C^* ) measurements obtained in the calibration study for the 10 electrodes used in the time-course studies together with the fitted values based on a linear model with interaction. Different symbols and colors correspond to different electrodes. Adapted with permission from [1]. Copyright 2017 American Chemical Society.

The specific study that motivated the problem considered in this paper was performed in the laboratory of one of the authors (Dr. P. Hashemi) at the Department of Chemistry and Biochemistry, University of South Carolina. The chemical concentration of interest is that of serotonin, a substance that that has been implicated in affective disorders such as depression, and the e.u.’s are mice (*in vivo*). The intervention performed on the mice is the administration of a pharmacological agent or drug. The type of data sets obtained from the study are shown in Figures 1 & 2. Figure 1 shows charge measurements over time for five different electrodes, with panels 1 and 2 showing results for the two intervention agents or drugs: pargyline and GBR 12909, respectively. Pargyline inhibits serotonin metabolism while GBR 12909 is a dopamine reuptake inhibitor. These drugs or agents inhibit serotonin metabolism and dopamine reuptake. Figure 2 presents the calibration data for ten electrodes, together with fitted mean response curves. The five electrodes used in the time-course study for each of the two drugs came from these 10 electrodes utilized in the calibration study. For instance, the electrodes used in the time-course experiment with pargyline were the electrodes labeled 2, 3, 4, 5, and 10; whereas, for GBR 12909 the electrodes were those labeled 1, 6, 7, 8, and 9.

The goal in this study is to estimate the mean concentration response function over time based on the time course study and the calibration study. Another aspect in the time-course study is that the drug intervention was administered after an initial no-drug period. Thus, another important goal is to determine the lag-time post administering the drug after which the drug takes effect, if indeed the drug has an effect. Discipline-specific details of this motivating and focused application has been previously published [1]. We point out the statistical methodology developed in this paper and the ideas contained herein have the potential of being useful in other situations where calibrated measurements are obtained [2].

## Mathematical setting

In this section we describe the postulated models for both the time-course study and the calibration study. For this purpose, let us suppose that we have a specimen from the e.u. We denote by *X*_*t*_ the concentration level of the chemical at time t for this specimen. We denote by *T* the time at which the intervention is performed, that is, the time of administering the drug intervention. We shall postulate the stochastic model

(1) $$X_{t}=\gamma_{0}+\gamma_{1}t+g\left(t-\left(T+\Delta \right)\right)+\varepsilon_{t},\;t\geq 0,$$

where ∆ is a lag-time and *g*(·) is a continuous function with *g*(*t*) = 0 for *t* ≤ 0. If smoothness is desired, we may impose the condition that *g*(·) is differentiable for *t* > 0. The time of intervention *T* is known, while the lag-time ∆, together with the regression coefficients *γ*_0_ and *γ*_1_, and the function g(·)are unknown. The error is assumed to be white noise, with *ε*_*t*_ having a normal distribution with mean zero and variance *τ*^2^, with *τ* also unknown. As discussed in section 1, the *X*_*t*_ could not be directly measured. If it could be directly measured and we possess the values of $X_{t}^{'}s$, then we could estimate the model parameters, and also infer the change point *T* + ∆, or the lag-time ∆.

To enable the determination of the chemical concentration levels $X_{t}^{'}s$, there are *K* possible electrodes that could be used for measuring the charge. At time *t*, *n*_*t*_ charge measurements using different electrodes will be taken. Thus, denoting by *Y* the charge and by *E* the electrode type, at time *t*, *n*_*t*_ pairs (*Y*_*tj*_, *E*_*tj*_), *j* = 1, 2, …, *n*_*t*_, are obtained. The measured charge is affected by the chemical concentration level and the electrode type. The relationship between the charge and the concentration and electrode type is given by

(2) $$Y=\left[\beta_{0}+\sum_{k=2}^{K}\xi_{k}I\left\{E=k\right\}\right]+\left[\beta_{1}+\sum_{k=2}^{K}\eta_{k}I\left\{E=k\right\}\right]X+\in$$

where *β*_0_, *β*_1_,*ξ*_2_,…*ξ*_*k*_,*η*_2_,…*η*_*k*_ are unknown, and ∈ has a normal distribution with mean zero and variance *σ*^2^, with *σ* unknown. The function *I*(·) is the indicator function, taking a 1(0)-value depending on whether the argument is true(false). This is a linear model that incorporates an interaction between the concentration and the electrode type [3,4].

In order to estimate the model parameters in model (2), a calibration study is performed. See [5] for some review of calibration methods. In this study, we followed the classic inverse regression approach. First, we regress the response variable, *Y*, on predictor *X*, and estimate linear regression coefficients using least-squares method; thereafter, the value of an unknown *X*, is to be estimated given an observation of *Y*, by subtracting the estimated intercept and dividing by the estimated slope [6–9]. Inference for the calibration parameters, is not trivial because of the presence of a normally distributed estimated slope in the denominator, which causes the inverse estimator to have infinite variance [10]. In this paper, we use Delta method to construct approximate confidence intervals for the calibration parameters. A more conservative confidence interval approach based on inverting simultaneous tolerance intervals was proposed by Scheffe [9] in literature. An alternative approach to the problem is referred to as reverse regression, when X’s are treated as the response and formally regressed on Y’s (even though the X’s are measured with negligible error). Krutchkoff [4] compared inverse and reverse regression using Monte Carlo simulations. Properties and limitations of the reverse estimators were studied by Williams [10] and Halperin [3].

In this calibration study, known levels of concentration are used, and the different electrodes are used to measure the charge. We denote by L the number of concentration levels, and these levels will be denoted by *x*_01_ < *x*_02_ < … < *x*_0*L*_. At concentration level *x*_0*l*_, all *K* electrode types are utilized, and for each electrode type, there are *m* charge measurements. Thus, for the *l*^*th*^ concentration level, there are *Km* observations, and for the *L* concentration levels, there are *N* = *LKm* charge measurements. The data could be summarized as in Table 1 and pictorially depicted as in Figure 2.

With *ξ*_1_ = *η*_1_ = 0, the linear models governing these charge measurements are given by

(3) $$Y_{ijl}=\left(\beta_{0}+\xi_{j}\right)+\left(\beta_{1}+n_{j}\right)x_{0i}+\in_{ijl}$$

for *i* = 1,…, *L*; *j* = 1,…, *K*; *l* = 1,…, *m*, and with the $\in_{ijl}^{'}s$ being independent and identically distributed (IID) and having a normal distribution with mean zero and variance *σ*^2^. These linear models could be fitted using object functions in a variety of statistical packages, such as the function lm or glm in the R statistical package [8].

## Estimating parameters

Instead of using the calibration data representation presented in Table 1, for purposes of describing more concisely the estimators of parameters, we denote by *Y* = (*Y*_1_,*Y*_2_,…,*Y*_*N*_)^*T*^ the *N* × 1 vector of charge values. The design matrix is *W*, which is an *N* × 2*K* matrix whose ith row is

$$W_{i}=\left[1,x_{i},v_{2i},\ldots ,v_{Ki},v_{2i}x_{i},\ldots ,v_{Ki}x_{i}\right]$$

with *x*_*i*_ the concentration level, and $v_{ji}=I\left\{E_{i}=j\right\}$, *j* = 2,…*K*, indicates whether the electrode type is *j*. With $\in ={\left(\in_{1},\in_{2},\ldots ,\in_{N}\right)}^{T}$ denoting the error vector, the linear model could be written via

(4) Y=Wθ+∈

where the 2*K* × 1 regression coefficient vector *θ* is

(5) $$\theta ={\left[\beta_{0},\beta_{1},\eta_{2},\ldots ,\eta_{K},\xi_{2},\ldots ,\xi_{K}\right]}^{T}.$$

In this model, $\in \sim N_{N}\left(0,\sigma^{2}I_{N}\right)$ where *I*_*N*_ is the *N* × *N* identity matrix.

The least-squares (LS) estimator of *θ*, which is also the maximum likelihood (ML) estimator, is given by (see, for instance, any linear theory book such as [6])

(6) $$\hat{\theta }={\left[W^{T}W\right]}^{-1}\left[W^{T}Y\right].$$

This is an unbiased estimator of *θ*. The error variance *σ*^2^is unbiasedly estimated by

(7) $${\hat{\sigma }}^{2}=\frac{1}{N-2K}{\left\|Y-W\hat{\theta }\right\|}^{2}=\frac{1}{N-2K}Y^{T}\left[I_{N}-H\right]Y$$

where $H=W{\left[W^{T}W\right]}^{-1}W^{T}$. An unbiased estimator of the covariance matrix of $\hat{\theta }$ is

(8) $$\hat{Cov\left(\hat{\theta }\right)}\equiv^{\wedge }\equiv {\hat{\sigma }}^{2}{\left[W^{T}W\right]}^{-1}.$$

The validation of the assumptions underlying this linear model could be performed graphically or via the global method in [7].

## Calibrated concentration estimators

The calibration problem will now be described. Suppose that, at a given time, we are given a charge measurement for a specific electrode: say, (*y*_0_, *e*_0_), where *e*_0_ is the electrode type and *y*_0_ is the charge on that specific electrode. More generally, suppose that *M* pairs of charge and electrode type are available at a given time: (*y*_01_, *e*_01_), (*y*_02_, *e*_02_),…, (*y*_0*M*_, *e*_0*M*_). Based on these observations, what is an estimate of the concentration level, and what is an approximate 100(1−*α*)% confidence interval for the concentration level?

## Calibration based on one pair (*y*_0_, *e*_0_)

Consider first just having one pair (*y*_0_, *e*_0_) of charge and electrode type observations. Denote by $x_{0}\equiv \left(y_{0},e_{0}\right)$ the concentration level that led to this charge value of *y*_0_ for electrode type *e*_0_. Based on the linear model relationship, *y*_0_ is a realization of the random variable

$$y_{0}=\left(\beta_{0}+\xi_{e0}\right)+\left(\beta_{1}+\eta_{e0}\right)x_{0}+\in_{0}$$

with the convention that *ξ*_1_ = *η*_1_ = 0 and $\in_{0}\sim N\left(0,\sigma^{2}\right)$. Solving for *x*_0_, we obtain

(9) $$X_{0}\left(y_{0},e_{0},\in_{0};\theta \right)=\frac{y_{0}-\left(\beta_{0}+\xi_{e0}\right)-\in_{0}}{\beta_{1}+\eta_{e0}}.$$

Of course, the error term ∈_0_ is not observable, but it has mean 0 and variance *σ*^2^. The expected value of $X_{0}\left(y_{0},e_{0},\in_{0};\theta \right)$, given (*y*_0_, *e*_0_), is

$$X_{0}\left(y_{0},e_{0},\in_{0};\theta \right)=E\left\{X_{0}\left(y_{0},e_{0},\in_{0};\theta \right)|\left(y_{0},e_{0}\right)\right\}=\frac{y_{0}-\left(\beta_{0}+\xi_{e0}\right)}{\beta_{1}+\eta_{e0}}$$

and its conditional variance is

$$Var\left\{X_{0}\left\{X_{0}\left(y_{0},e_{0},\in_{0};\theta \right)|\left(y_{0},e_{0}\right)\right\}\right\}=\frac{\sigma^{2}}{{\left(\beta_{1}+\eta_{e0}\right)}^{2}}.$$

Consequently, a plausible estimator of *x*_0_(*y*_0_, *e*_0_) is

(10) $${\hat{x}}_{0}\left(y_{0},e_{0};\hat{\theta }\right)=g_{e0}\left(y_{0};\hat{\theta }\right)\equiv \frac{y_{0}-\left({\hat{\beta }}_{0}+{\hat{\xi }}_{e0}\right)}{{\hat{\beta }}_{1}+{\hat{\eta }}_{e0}}.$$

This is the calibrated estimate of the concentration when given a charge value of *y*_0_ obtained using the electrode type *e*_0_. By the Delta-Method [2], this will be approximately unbiased for the expectation of *x*_0_(*y*_0_, *e*_0_). We seek an approximation of its variance by using the delta method. For *j* = 1, 2,…, *K*, define the gradients

$$b_{j}\left(y_{0};\theta \right)=\frac{\partial }{\partial \theta }\left[\frac{y_{0}-\left(\beta_{0}+\xi_{j}\right)}{\beta_{1}+\eta_{j}}\right].$$

By the Delta-Method, an estimate of the variance of ${\hat{x}}_{0}\left(y_{0},e_{0}\right)$ is

(11) $$Var\left[\hat{\hat{x}\left(y_{0},e_{0};\hat{\theta }\right)}\right]\equiv V_{0}^{2}\left(y_{0},e_{0}\right)=\;\;\;\;{\hat{\sigma }}^{2}\left[b_{e0}{\left(y_{0},\hat{\theta }\right)}^{T}{\left[W^{T}W\right]}^{-1}b_{e0}\left(y_{0},\hat{\theta }\right)+\frac{1}{{\left({\hat{\beta }}_{1}+{\hat{\eta }}_{e0}\right)}^{2}}\right].$$

An approximate 100(1−*α*)% confidence interval for the concentration level, having observed a charge value of *y*_0_ using electrode type *e*_0_, is given by

(12) $$\left[{\hat{x}}_{0}\left(y_{0},e_{0};\hat{\theta }\right)\pm \left(t_{N-2K;\alpha /2}\right)V_{0}\left(y_{0},e_{0}\right)\right],$$

Where $t_{N-2K;\alpha /2}$ is the (1− *α/*2)*th* quantile of a t-distribution with degrees-of-freedom *N* − 2*K*.

## Calibration based on many pairs (*y*_0_, *e*_0_)

Next we consider the situation where several charge measurements are taken using possibly different electrode types. Let

$$\left(y_{0},e_{0}\right)=\left\{\left(y_{0m},e_{0m}\right):m=1,2,\ldots ,M\right\}.$$

Here *y*_0*m*_ is the *mth* charge measurement which is obtained using electrode type *e*_0*m*_. From the preceding subsection, based on this particular measurement we obtain an estimate of the concentration level, given by

$${\hat{x}}_{0}\left(y_{0},e_{0};\hat{\theta }\right)$$

which has an approximate estimated variance of $V_{0m}^{2}\left(y_{0m},e_{0m}\right)$ whose expression is obtained via (11). The *M* estimates of the concentration level obtained for each of the elements in (*y*_0_, *e*_0_) will not be independent of each other, owing to the fact that they will all depend on $\hat{\theta }$. In fact, through the Delta-Method, we could obtain their approximate covariance matrix. However, for the sake of simplicity and practicality, we ignore the dependence among these M estimates. Under this simplified assumption, whose appropriateness will be examined later through simulation studies, we could then combine the M estimates by simply taking into account the possibly varying estimates of their variances. We recall the following well-known distribution theory result, which is easily proved using a Lagrange multiplier minimization approach.

### Theorem 1

Let *W*_1_,*W*_2_,…,*W*_*m*_ be independent random variables with common mean *μ* and respective variances $\tau_{1}^{2},\tau_{2}^{2},\ldots ,\tau_{m}^{2}$. Among all linear combinations $\sum_{l=1}^{m}c_{l}W_{l}$ with $\sum_{l=1}^{m}c_{l}=1$, so that the mean of the linear combination is still *μ*, the one with the smallest variance coincides with the choice of coefficients given by

$$c_{l}^{*}=\frac{\left(1/\tau_{1}^{2}\right)}{\sum_{l=1}^{m}\left(1/\tau_{1}^{2}\right)},\;\;l=1,2,\ldots ,m.$$

The variance of this optimal linear combination is

$$V=\left[\sum_{l=1}^{m}c_{l}^{*}W_{l}\right]={\left[\frac{1}{m}\sum_{l=1}^{m}\frac{1}{\tau_{1}^{2}}\right]}^{-1}.$$

Using this result, we obtain our combined estimate of the concentration level from the *M* estimates via

(13) $${\hat{x}}_{0}\left(y_{0},e_{0};\hat{\theta }\right)=\sum_{m=1}^{m}{\hat{c}}_{m}{\hat{x}}_{0}\left(y_{0m},e_{0m};\hat{\theta }\right),$$

where the weights are given by

(14) $${\hat{c}}_{m}=\frac{\left[1/V_{0m}^{2}\left(y_{0m},e_{0m}\right)\right]}{\sum_{q=1}^{M}\left[1/V_{0q}^{2}\left(y_{0q},e_{0q}\right)\right]},m=1,2,\ldots M.$$

The approximate variance of ${\hat{x}}_{0}\left(y_{0},e_{0};\hat{\theta }\right)$ is given by

(15) $$V_{0}^{2}\left(y_{0},e_{0}\right)={\left[\frac{1}{M}\sum_{m=1}^{M}\frac{1}{V_{0}^{2}\left(y_{0m},e_{0m}\right)}\right]}^{-1},$$

As a consequence, an approximate 100(1− *α*)% confidence interval for the concentration level, having observed (*y*_0_, *e*_0_), is given by

(16) $$\left[{\hat{x}}_{0}\left(y_{0},e_{0};\hat{\theta }\right)\pm \left(t_{N-2K;\alpha /2}\right)V_{0}\left(y_{0},e_{0}\right)\right].$$

## Time-course study

As mentioned in section, 1 the main goal of the study is to determine the behavior of the concentration of the chemical over a period of time, where at some point *T* during the monitoring period, a drug intervention is performed. We suppose that over the period [0,T*] charge measurements using possibly different electrode types are performed at specified times 0 ≤ *t*_1_ < *t*_2_, < *t*_3_ <…< *t*_*L*_ ≤T*. The charge and electrode observations at time *t*_1_ are given by (*y*_0*l*_, *e*_0*l*_). Based on these observation vectors, we find an estimate of the concentration level at time *t*_1_ to be

$${\hat{x}}_{0l}\left(y_{0l},e_{0l}\right),$$

together with its estimate of its variance $V_{0l}^{2}\left(y_{0l},e_{0l};\hat{\theta }\right)$. For each time *t*_*l*_, we could also construct the approximate 100(1− *α*)% confidence interval for the concentration value given by

$$\left[{\hat{x}}_{0l}\left(y_{0l},e_{0l};\hat{\theta }\right)\pm \left(t_{N-2K;\alpha /2}\right)V_{0l}\left(y_{0l},e_{0l}\right)\right].$$

Note, however, that these confidence intervals are not adjusted for multiplicity. On the other hand, based on the time course data and the resulting concentration estimates at each of the times of observations, given by

$$\left\{\left(t_{l},{\hat{x}}_{0l}\left(y_{0l},e_{0l};\hat{\theta }\right),V_{0l}\left(y_{0l},e_{0l}\right)\right):l=1,2,\ldots ,L\right\},$$

we could fit an appropriate regression curve that takes into account the possibly differing variability of the ${\hat{x}}_{0l}{\left(y_{0l},e_{0l};\hat{\theta }\right)}^{'}s$. One of the simplest models that could be fitted to this data, under the hypothesis that upon the drug intervention at time *T* the concentration curve should change after a lag-time of ∆, specifies that

(17) $${\hat{x}}_{0l}=\omega_{0}+\omega_{1}t_{l}+k_{1}U_{l}+k_{2}U_{l}^{2}+V_{0l\varepsilon l},$$

and

$$U_{l}=\max\left(0,t_{1}-\left(T+\Delta \right)\right),l=1,2,\ldots ,L,$$

and *ε*_*l*_ has mean zero and variance *τ*^2^. Note that the *τ*^2^ in this model is not the same as the *τ*^2^ in the time-course model in (1). Here, the time of intervention *T* is known, whereas the lag-time ∆ is not known. For a specified ∆, this model is easily fitted using the lm command in R with the weights option enabled, which performs a weighted linear regression fit [8]. The coefficient of determination could be obtained and denoted by *R*^2^(∆). The coefficient of determination could be plotted with respect to possible values of ∆. The value of ∆ that maximizes this coefficient of determination *R*^2^(∆) is a plausible estimate for the lag-time ∆. Thus, a possible estimator of the lag-time is

(18) $$\hat{\Delta }=\arg\underset{\Delta }{\max}R^{2}\left(\Delta \right).$$

This could be computed by fitting the model over a sequence of values of ∆ and searching for the value of ∆ that maximizes *R*^2^(∆). This computational method is the approach we implemented in this paper. The confidence interval described above at a given time point is appropriate if we only have data at one time point. However, in the time-course study, we actually obtain a series of concentration estimates for each of the time points considered. These pairs of time and concentration estimates are then used to estimate the time course model parameters. In the process of fitting the linear-parabolic curve, we could then also obtain pointwise confidence intervals for the concentration value at each of the time points, where we utilize the estimate of the error standard deviation. We surmise that the resulting point-wise confidence intervals is a better indicator of where the concentration values are. In the real-data application of the procedure in section 6, this point-wise confidence interval approach will be presented.

## A real-data illustration

This section provides a more detailed description of the statistical methods performed in the data analysis in some portions of paper [1]. The data sets were obtained in the Hashemi laboratory at University of South Carolina. We limit our consideration to the intervention drug pargyline, which was hypothesized to have an impact on the concentration level of serotonin, in contrast to the intervention drug GBR 12909. Figures 1–2 present the time-course data and the fitted linear models. These figures are adapted with permission from [1] and are copyrighted from American Chemical Society. In the next section, we then present simulation studies to provide us some ideas on the properties of this procedure.

A time-course study was performed leading to the data set with charge measurements over time (from 0 minutes to 121 minutes), with the intervention drug pargyline (10 mg/kg) administered at 60 minutes, via interperitoneal injection. The time-course data with pargyline as the intervention drug is depicted in the first panel of Figure 1. The different symbols correspond to the 5 different electrodes (out of 10 possible electrodes) used in measuring the charges. A calibration study was also performed where, for known concentration levels, charge measurements were obtained for each of 10 possible electrodes. The charges of each electrode are measured under three concentration values (25, 50, and 100 nM) each with 4 replicates. The data set obtained from this calibration study are the solid points in Figure 2.

A linear model, as described in (2), was fitted using the calibration data. The fitted linear models, using the calibration data, for each electrode type are depicted as the lines in Figure 2 for each of the 10 electrodes. A summary of the estimated model parameters are in Table 2. These linear models with interaction terms provide excellent fits to the observed calibration data with coefficient of determinations all above 99%. The interaction effects are all significant.

**Figure 3:**
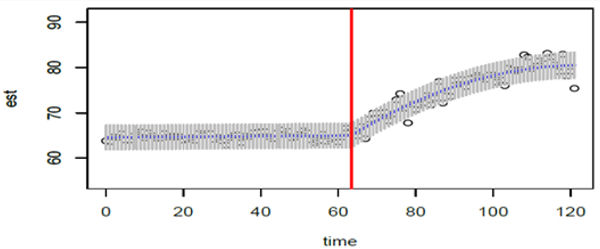
Plot of the concentration over time with the fitted linear-parabolic model and point-wise confidence intervals for the intervention drug pargyline.

Using these fitted linear models, given the charge measurements at each time point from the time course study, estimates of the concentration levels at each time point were obtained. This procedure yielded pairs of values of time and concentration estimates which are the solid points in Figure 3. A functional continuous model, as described in Section 5, was fitted to these pairs of time and concentration values using weighted regression via the lm command in the R environment. The estimate of the time-lag ∆ after which the drug takes effect is $\hat{\Delta }=3.32\text{minutes}$. As mentioned earlier, this estimate was obtained by maximizing the coefficient of determination with respect to the possible values of ∆ [see discussion prior to (18)]. The resulting fitted linear-parabolic model whose equation is given in (18) is shown in Figure 3.

(19) $$\hat{C}\left(t\right)=64.584+0.0063t+0.5163U\left(t\right)-0.0044U{\left(t\right)}^{2}$$

where *U* (*t*) = max (0, 63.32 – *t*). This plot is depicted together with the concentration estimates at each time point, which are the open circles in the plot. Point-wise confidence interval at each time point is also included in the plot. These 95% point-wise confidence intervals were constructed when the functional model was fitted to the pairs of time and concentration values using the predict. lm command in R. Details pertaining to the fitted functional relationship between time and concentration are summarized in Table 3. Observe that the coefficient for the time effect is not significantly different from (*p* = .429) which is to be expected since without drug intervention the serotonin concentration is not expected to change. We also mention that the final fitted model has an *R*^2^ equal to 95.59%, indicating an excellent fit of the linear-parabolic model for relating concentration to time for this study.

## Simulation studies

In order to study the properties of the procedure described above for estimating the lag-time parameter ∆, we performed a computer simulation study. All simulation programs were coded in *R* and function objects in *R* such as lm, rnorm, etc. were utilized. The main purpose of this study was to determine if we are able to effectively estimate the lag-time parameter ∆ using a time-course data and a calibration data. In the simulation, we generate the time-course data using the model

(20) $$x_{t}=\gamma_{0}+\gamma_{1}t+k_{1}\max\left(0,t-\left(T+\Delta \right)\right)+k_{2}{\left[\max\left(0,t-\left(T+\Delta \right)\right)\right]}^{2}+\tau Z_{t}^{\left(1\right)}$$

where *γ*_0_ = 20, *γ*_1_ = .25, *k*_1_ = 10, *k*_2_ = −.12, *T* = 60, *τ* = 10 and with $Z_{t}^{\left(1\right)'}s$ variables. The time index took values in {0,1, 2,…,120}. Thus, for each *t* ∈{1, 2,…,120} an *x*_*t*_ was generated according to this model.

At time *t* and for the generated *x*_*t*_ – *value*, a charge measurement was undertaken using three electrode types: *m* = 1, 2, 3. The charge value was generated according to the model

(21) $$Y_{m}=\left(\beta_{0}+\xi_{m}\right)+\left(\beta_{1}+\eta_{m}\right)x_{t}+Z_{tm}^{\left(2\right)}$$

Where $Z_{tm}^{\left(2\right)'}s$ are IID random variables. The parameter values were set to *β*_0_ = 1, *β*_1_ = 1.5, *ξ* = (0,1,2), *η* = (0,.5,1.5). Thus, this leads to the ‘observed time-course data’ given by

$$\left\{\left(t,y_{tm}\right),m=1,2,3;t=0,1,2,\ldots 120\right\}.$$

A calibration experiment was also simulated. In this calibration experiment, five specific concentration values *x* ∈ {0,10, 25, 50,100} were utilized. At each of these concentration values, five charge values were generated for each of the three electrode types according to the model

$$Y_{jmk}=\left(\beta_{0}+\xi_{m}\right)+\left(\beta_{1}+\eta_{m}\right)x_{j}+Z_{jmk}^{\left(3\right)},k=1,2,\ldots ,5,$$

where the parameter values are the same as in the preceding model, and *x*_1_ = 0, *x*_1_ = 10, *x*_3_ = 25, *x*_4_ = 50, *x*_5_ = 100 and $Z_{jmk}^{{\left(3\right)}^{\prime }}s$ are IID *N* (0,1) random variables.

On the basis of the time-course data

$$\left\{\left(t,y_{tm}\right),m=1,2,3;t=0,1,2,\ldots ,120\right\}$$

and the calibration data

$$\left\{\left(x_{j},y_{jmk}\right),j=1,2,\ldots ,5;m=1,2,3;k=1,2,\ldots ,5\right\},$$

the goal is to see if we could reasonably recover the signal function given by

(22) $$g\left(t\right)=E\left\{X_{t}\right\}=\gamma_{0}+\gamma_{1}t+k_{1}\max\left(0,t-\left(T+\Delta \right)\right)+k_{2}{\left[\max\left(0,t-\left(T+\Delta \right)\right)\right]}^{2}$$

and obtain good estimates of the model parameters using the procedure described in the preceding sections, in particular to obtain a reasonable estimate of ∆.

**Figure 4:**
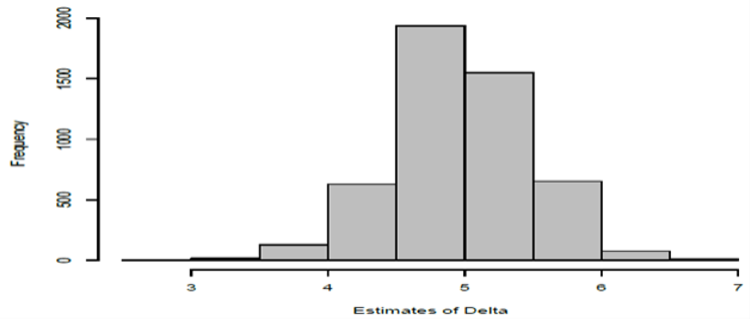
Frequency histogram of the estimates of from the simulation study. The true value was and there were 5000 replications.

**Figure 5:**
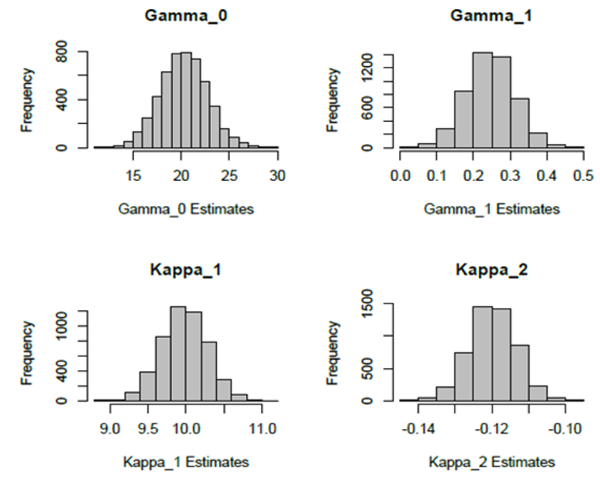
Frequency histograms of the 5000 estimates of the model parameters obtained from the simulation study. The true values of the parameters are *γ*_0_ = 20.0, *γ*_1_ = 0.25, *k*_1_ = 10.0, *k*_2_ = −0.12.

We performed the above-described simulation experiment using 5000 replications. The estimates of ∆ are provided in the frequency histogram in Figure 4. In searching for the optimizing ∆, we used increments of 0:1, so that the possible values of $\hat{\Delta }$ contains only one decimal digit. The mean of these ${\hat{\Delta }}^{\prime }s$ is 5:03828 with a standard error of 0:4990. Thus, this mean is quite close to the true value of ∆ which is 5.0. Regarding the other model parameters pertaining to the time-course portion, we summarize the results in Table 4. Histograms of these 5000 estimates for the four model parameters are depicted in Figure 5. Observe that the means of these estimates for each of the regression parameters are close to their true values.

**Figure 6:**
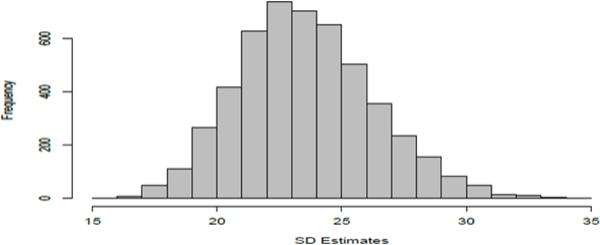
Histogram of the 5000 estimates of the standard deviation of the error term of the time-concentration linear-parabolic regression model.

Another parameter in the time-course model is *τ*, which is the standard deviation of the error component. When we fit the linear-parabolic model on the time-course and calibration data, an estimate of the error standard deviation is also obtained. However, this is not an estimate of *τ* since this estimates a larger value than *τ* owing to the additional error contamination arising from the calibration study and the charge measurements at each time and for each electrode type. For instance, in the simulation study, the histogram of the 5000 estimates of the standard deviation of the error term provided in Figure 6 all exceed the true value of *τ* = 10. The mean of these standard deviation estimates is 23.56979 with a standard deviation of 2.723542. On the basis of this modest simulation study, the statistical procedure developed in earlier sections using the calibration and time-course data sets appears successful in recovering the functional relationship between time and concentration and is also able to infer properly about the lag-time at which the drug starts to become effective.

In order to further study the properties of the statistical procedure on the real data, we performed another simulation study using the estimated model parameters based on the data from the Hashemi laboratory. The time course data was generated using model (20), with *T* = 60, ∆ = 3.3, *τ* = 0.3, and with $Z_{t}^{\left(t\right)'}s$ IID *N*(0,1) variables. The values of other parameters were set to be the same as the estimated ones in Table 3. The time index took values in {0,1,2,…121}. Charge measurements were obtained using five electrodes *m* = 2, 3, 4, 5,10. The charge values were generated according to the model (21) with parameter values

$$\beta_{0}=0.6950,\beta_{1}=0.0116;$$

ξ=(2.1707,2.6107,2.4622,1.42622,1.4289,1.3953);

η=(0.0270,−0.0054,0.0152,0.0082,0.0024),

and

$$Z_{tm}^{\left(2\right)}\;\text{and IID}\;N\left(0,0.095^{2}\right).$$

These correspond to the estimates of the model parameters in section 6. To simulate the calibration data, we generated charge values with concentration taking values *x* ∈{25, 50,100} for 10 electrodes, i.e. *m* = 1, 2,…,10, according to

$$Y_{jmk}=\left(\beta_{0}+\xi_{m}\right)+\left(\beta_{1}+\eta_{m}\right)x_{j}+Z_{jmk}^{\left(3\right)},k=1,2,3,4,$$

where the parameter values were set identical to the estimated values in Table 2, and with *x*_1_ = 25, *x*_2_ = 50, *x* = 100, and $Z_{jmk}^{\left(3\right)\prime }s$ being IID from a *N*(0, 0.095^2^) distribution. Using the time-course data and the calibration data, it is of interest to see if we could reasonably recover the signal function in (22) and also obtain good estimates of the model parameters.

**Figure 7:**
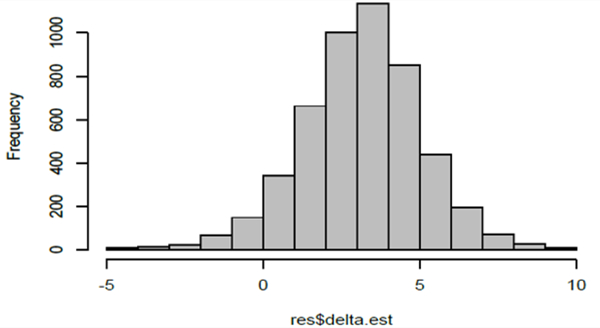
Frequency histogram of the estimates of from the simulation study with 5000 replications. The true value was ∆ = 3.3.

**Figure 8:**
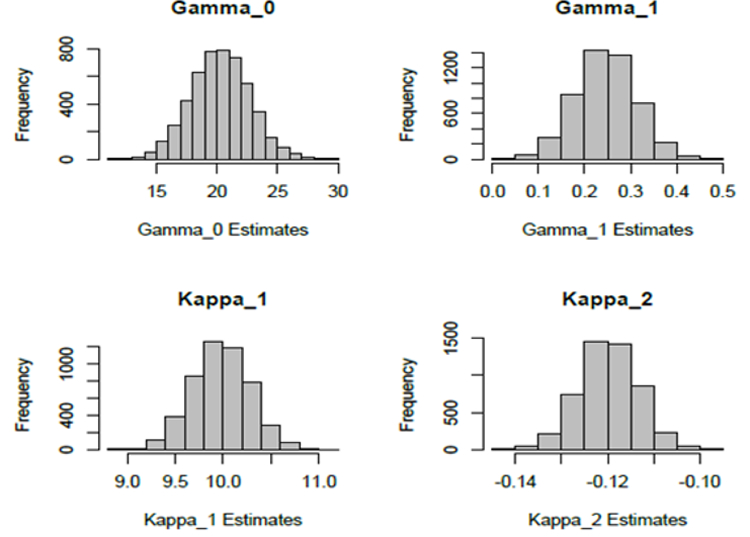
Frequency histograms of the 5000 estimates of the model parameters obtained from the simulation study. The true values of the parameters are *γ*_0_ = 64.5839, *γ*_1_ = 0.006311, *k*_1_ = 0.5163, *k*_2_ = −0.004390.

The resulting 5000 estimates of ∆ are provided in the frequency histogram in Figure 7. The mean of these 5000 ${\hat{\Delta }}^{\prime }s$ is 3:3008 with a standard error of 1:9341. The mean is indeed very close to the true value which is ∆ = 3.3, indicating the possible unbiasedness of the estimator, though of course a simulation study could not establish this theoretical property. Note, however, that there were some negative estimates that arose and this could be a consequence of a wrong (we hypothesize larger values) specification of the error variances in the models. Details of the estimates for the other parameters are summarized in Table 5. Histograms of the estimates for these four model parameters are depicted in Figure 8.

## Concluding Remarks

Motivated by a study in an analytical chemistry laboratory dealing with the concentration of the chemical substance serotonin, we developed in this paper a statistical procedure for estimating the functional relationship between time and concentration level of a substance, together with the change point, based on a time-course study that measures a concentration-surrogate variable (the charge in this application) and data from a calibration study. The novel aspect of this procedure is the presence of several measuring electrodes which have their unique behaviors when utilized to measure the surrogate variable (the charge in application). Simulation studies were performed to examine the properties of the proposed procedures, and the simulation results indicate that the procedure is able to reasonably recover the signal function and also the change-point in the signal function.

The procedure is also applied to the time-course data and the calibration data from the focus application obtained in the analytical chemistry experiment. Further theoretical studies and simulations will be desired to examine more carefully the procedure especially when applied to more complex settings such as when the *g* (·) portion in the signal function in equation (1) is not parabolic or when it is non-parametrically specified. Of interest for further research are situations where the error distributions in the regression models are not normally-distributed, which may entail the use of non-parametric regression estimation methods.

## Figures and Tables

**Table 1:** Format of the calibration data from the calibration study.

Electrode Type	Concentration Levels			
	*x*_01_	*x*_02_	…	*x*_0*L*_
1	$Y_{111,\ldots ,Y_{11m}}$	$Y_{211,\ldots ,Y_{11m}}$	…	$Y_{L11,\ldots ,Y_{L1m}}$
2	$Y_{121,\ldots ,Y_{12m}}$	$Y_{221,\ldots ,Y_{22m}}$	…	$Y_{L21,\ldots ,Y_{L2m}}$
፧	፧	፧	፧	፧
*K*	$Y_{1K1,\ldots ,Y_{1Km}}$	$Y_{2K1,\ldots ,Y_{2Km}}$	…	$Y_{LK1,\ldots ,Y_{LKm}}$

**Table 2:** Estimates, including their estimated standard errors (SE), of the parameters of the calibration model. Corresponding p-values are provided in the last column.

Coefficient	Estimates	SE of Estimates	p-value
*β*_0_	0.6949750	0.0581590	< 2 × 10^−16^
*β*_1_	0.0115531	0.0008793	< 2 × 10^−16^
*ξ*_2_	2.1706500	0.0822493	< 2 × 10^−16^
*ξ*_3_	2.6106750	0.0822493	< 2 × 10^−16^
*ξ*_4_	2.4621500	0.0822493	< 2 × 10^−16^
*ξ*_5_	1.4289000	0.0822493	< 2 × 10^−16^
*ξ*_6_	0.9786500	0.0822493	< 2 × 10^−16^
*ξ*_7_	−0.2215625	0.0822493	0.00829
*ξ*_8_	1.1935250	0.0822493	< 2 × 10^−16^
*ξ*_9_	1.0626500	0.0822493	< 2 × 10^−16^
*ξ*_10_	1.395275	0.0822493	< 2 × 10^−16^
*η*_2_	0.027039	0.0012435	< 2 × 10^−16^
*η*_3_	−0.0053756	0.0012435	3.64 × 10^−05^
*η*_4_	0.0151561	0.0012435	< 2 × 10^−16^
*η*_5_	0.0082419	0.0012435	1.74 × 10^−09^
*η*_6_	0.0184476	0.0012435	< 2 × 10^−16^
*η*_7_	−0.0028579	0.0012435	0.02363
*η*_8_	0.0089354	0.0012435	1.22 × 10^−10^
*η*_9_	0.0379233	0.0012435	< 2 × 10^−16^
*η*_10_	0.0023726	0.0012435	0.05926

**Table 3:** EEstimates, together with estimates of the standard error (SE), of the coefficients in the time-course model. Corresponding p-values are provided in the last column.

Regressor	Estimate	SE of Estimate	p-Value
1	64.583863	0.315068	< 2 × 10^−16^
*t*	0.006311	0.007949	0.429
*U*(*t*)	0.516323	0.033507	< 2 × 10^−16^
*U*(*t*)^2^	−0.004390	0.000540	4.86 × 10^−13^

**Table 4:** Means and standard deviations of the 5000 estimates obtained in the simulation study of the four model parameters *γ*_0_, *γ*_1_, *k*_1_, *k*_2_ in the time-course portion of the model. The true parameter values are provided in the second column.

Parameter	True Value	Mean of Estimates	SD of Estimates
*γ*_0_	20.00	20.28285	2.45459
*γ*_1_	0.25	0.24523	0.06517
*k*_1_	10.00	9.98088	0.29866
*k*_2_	−0.12	−0.11980	0.00629

**Table 5:** Means and standard deviations of the 5000 estimates obtained in the simulation study of the four model parameters *γ*_0_, *γ*_1_, *k*_1_, *k*_2_, in the time-course portion of the model. The true parameter values are provided in the second column.

Coefficient	True Value	Mean of Estimates	SD of Estimates
*γ*_0_	64.5839	64.5718	0.6921
*γ*_1_	0.006311	0.005909	0.01278
*k*_1_	0.5163	0.5169	0.05266
*k*_2_	−0.004390	−0.004436	0.001013

## Abbreviations:

EU: Experimental Unit
IID: Identically Distributed
LS: Least-Squares
ML: Maximum Likelihood
